# National assessment of obstetrics and gynecology and family medicine residents’ experiences with CenteringPregnancy group prenatal care

**DOI:** 10.1186/s12884-023-06124-0

**Published:** 2023-11-21

**Authors:** Jean Marie Place, Kristin Van De Griend, Mengxi Zhang, Melanie Schreiner, Tanya Munroe, Amy Crockett, Wenyan Ji, Alexandra L. Hanlon

**Affiliations:** 1 Department of Nutrition and Health Science, Public Health, Ball State University, Office 546, 1613 W. Riverside Ave, Muncie, IN USA; 2 Department of Health Sciences, Community and Public Health, Idaho State University, Pocatello, ID USA; 3 Health Systems and Implementation Science, Virginia Tech Carilion School of Medicine, Roanoke, USA; 4 IU Health Family Medicine Residency Center, Muncie, IN USA; 5 Quality and Special Initiatives, Centering Healthcare Institute, Boston, MA USA; 6 Department of Obstetrics and Gynecology, Prisma Health, Greenville, SC USA; 7 Department of Statistics, Center for Biostatistics and Health Data Science, Virginia Tech, Roanoke, VA USA; 8 Department of Statistics, Biostatistics and Health Data Science, Virginia Tech, Roanoke, VA USA

**Keywords:** CenteringPregnancy, Group prenatal care, Resident education, Family medicine, Obstetrics and gynecology

## Abstract

**Objective:**

To examine family medicine (FM) and obstetrician-gynecologist (OB/GYN) residents’ experiences with CenteringPregnancy (CP) group prenatal care (GPNC) as a correlate to perceived likelihood of implementing CP in future practice, as well as knowledge, level of support, and perceived barriers to implementation.

**Methods:**

We conducted a repeated cross-sectional study annually from 2017 to 2019 with FM and OB/GYN residents from residency programs in the United States licensed to operate CP. We applied adjusted logistic regression models to identify predictors of intentions to engage with CP in future practice.

**Results:**

Of 212 FM and 176 OB/GYN residents included in analysis, 67.01% of respondents intended to participate as a facilitator in CP in future practice and 51.80% of respondents were willing to talk to decision makers about establishing CP. Both FM and OB/GYN residents who spent more than 15 h engaged with CP and who expressed support towards CP were more likely to participate as a facilitator. FM residents who received residency-based training on CP and who were more familiar with CP reported higher intention to participate as a facilitator, while OB/GYN residents who had higher levels of engagement with CP were more likely to report an intention to participate as a facilitator.

**Conclusion:**

Engagement with and support towards CP during residency are key factors in residents’ intention to practice CP in the future. To encourage future adoption of CP among residents, consider maximizing resident engagement with the model in hours of exposure and level of engagement, including hosting residency-based trainings on CP for FM residents.

**Supplementary Information:**

The online version contains supplementary material available at 10.1186/s12884-023-06124-0.

## Introduction

CenteringPregnancy (CP) is a clinical, comprehensive, evidence-based approach to prenatal care (GPNC) that has reduced racial disparities in preterm birth among African American women relative to white women, contributed to higher birth weight infants in a general population, and improved psychosocial outcomes, including better prenatal knowledge and perceived preparation for labor and delivery, compared to traditional prenatal care [1–10]. CenteringPregnancy is based in groups rather than one-on-one provider-patient appointments. Eight to ten women with similar gestational ages receive the recommended schedule of 10 prenatal care visits together for 90 to 120 min per visit and engage in health assessment, facilitative discussion, and interactive activities about pregnancy and the postpartum.

In a healthcare delivery context shaped by widening ratios of family physicians to number of patients [11, 12], training family medicine (FM) residents in CP may be a way to improve healthcare by expanding access to prenatal care [11–13] while potentially reducing costs to the healthcare system [14]. Possibly just as important, both patients [8, 15] and providers [16, 17] report high satisfaction with CP which may help prevent professional burnout. Nationally, GPNC has been included as part of the curriculum for both FM [18] and obstetrics and gynecology (OB/GYN) residency programs because of its ability to integrate the teaching of obstetric medical knowledge with important elements of professionalism and patient-centered communication skills [18–21]. Residents involved in CP have reported increases in competency-based skills in facilitation and team collaboration [22]. FM residents who participate in CP during residency have been found to be more likely to include obstetrics in their practice after residency, and to go on to obstetric fellowships after residency [18], suggesting the importance of CP in maintaining a wide scope of practice for FM physicians.

The current study examines a national sample of residency settings to elucidate the drivers behind intention to establish CP post-residency. Thus, the purpose of the study is to conduct a national assessment of FM and OB/GYN residents’ experience with CP as a correlate to their perceived likelihood of implementing CP in future practices and their knowledge, level of support, and perceived barriers toward implementing this model of care.

## Materials and methods

### Design and setting

We conducted a repeated, cross-sectional study at three consecutive timepoints among residency programs that had an existing CP program site approval by the Centering Healthcare Institute, the non-profit CP accrediting body. Data collection occurred during approximately four weeks per year for three years from 2017 to 2019. To capture different levels of engagement with CP, we sent annual invitations to participate in the study to residency programs in late June. This is approximately the period when new residents are incoming and graduating residents are outgoing. We invited participation from FM (PG Year 1–3) and OB/GYN residents (PG Year 1–4) from residency programs with CP program site approval (379 in 2017 and 2018; 383 from 2019) from a total of 33 states in the United States. These sites hold formal CP groups based on their site approval from the Centering Healthcare Institute, but they vary regarding whether and/or when residents are required to participate in the groups. The study received ethics approval by the IU Health Ball Memorial Hospital institutional review board.

### Participants and procedure

An invitation letter (accompanied by a letter of support from the Centering Healthcare Institute), consent form, and link to the online survey was sent via email to individuals responsible for CP at each facility, who were then asked to electronically forward the material to all FM and/or OB/GYN residents at the facility. Three follow-up, reminder emails were sent, up to a month following the initial invitation.

### Instruments

The multi-domain survey was developed by the researchers and assessed for face and content validity by a panel of seven reproductive health experts. Experts were sent a link to the survey and asked to assess the ‘worthiness’ of each question by indicating whether they thought the item was essential to the survey, helpful but not essential, or not helpful nor appropriate. Experts were asked to explain their response for each item in a comment box. Prior to data collection among FM and OB/GYN residents, we conducted cognitive interviewing on the instrument with two FM residents to refine survey questions. The final survey included demographic information and assessed five primary domains.

#### Outcome measures: intention to engage with CenteringPregnancy in future practice

The outcome measures were comprised of two separate items: 1) intention to participate as a group facilitator in future practice if CP is established and 2) intention to talk to decision makers in future practice about establishing CP if it is not currently being practiced. Both items had five-point Likert response options from strongly agree to strongly disagree. The variable was dichotomized as “did not agree” (neither agree nor disagree, disagree, strongly disagree) or “agreed” (strongly agree, agree).

#### Predictors: knowledge & familiarity of CenteringPregnancy

Knowledge of the purpose of CP was measured by one item asking respondents to choose from a multiple-choice question about the purpose of CP. The answer was scored as 1 (have never heard about it /do not know what it is), 2 (a support group/an education group), and 3 (group-based, comprehensive prenatal care), which was the correct answer. Self-reported familiarity was measured by one item—participants’ reported familiarity of CP—with six-point Likert response options (1 = not familiar to 6 = very familiar). The variable was recategorized into not familiar, somewhat familiar, familiar, and very familiar.

#### Predictors: experience with CenteringPregnancy

Experience with the CP model consisted of three items that address: 1) level of engagement with CP; 2) types of engagement with CP; and 3) clinical and didactic hours exposed to CP. The level of engagement with CP was measured by counting the number of items participants were exposed to including continuing education through conferences or in-service training off-site, trainings held at the residency center, trainings held at the CenteringHealthcare Institute, classroom study and formal coursework, media attention (newspapers, radio, tv, social media, etc.), journal article or scholarly literature, or other source. An item measuring the number of hours spent in any of the seven listed sources of exposure to CP was used to measure participants’ hours of exposure. Response options included less than 5 h, 5–10 h, 10–15 h, or 15 or more hours. The variable was scored as less than 5 h, between 5 and 15 h, or more than 15 h.

#### Predictors: level of support for CenteringPregnancy

Level of support for CP consisted of nine items related to respondents’ perceptions of quality of care, patient education, patient-centered attention, and social support, all with five-point Likert response options (1 = strongly agree to 5 = strongly disagree). Scores were summed such that higher scores reflected less support for CP.

#### Predictors: anticipated barriers

Anticipated barriers to establishing CP or being a physician at a practice that offers CP were assessed using seven questions. They included perceived challenges with productivity goals, time management, lack of institutional support or appropriate training, continuity and quality of care, and cost, using a five-point Likert scale (1 = strongly agree to 5 = strongly disagree). A higher score reflected fewer anticipated barriers.

#### Covariates

Potential confounders in the association between predictors and outcome variables were identified from previous publications and were thus controlled in the adjusted models [18, 23, 24]. These variables include year of participation (1 = 2017 (ref), 2 = 2018, 3 = 2019), sex (1 = Male (ref), 2 = Female, 3 = Intersex), age (1 = 30 and less (ref), 2 = more than 30), race/ethnicity (1 = White (ref), 2 = Non-White (Hispanic/Latino, Black/African American, Native American/American Indian, Asian/Pacific Island, or other race/ethnicity), 3 = Multiracial). All of the above variables were treated as categorical variables in data analyses. Residency year (year 1–4), however, was included in analyses as a continuous variable.

### Statistical analyses

Variables of interest, including demographic and program characteristics (sex, age, race/ethnicity, residency year), potential predictors (types and level of engagement with CP, hours exposed to CP, knowledge and self-reported familiarity of CP, level of support for CP, and anticipated barriers of CP) and outcomes (dichotomized intentions to engage with CP), were summarized between FM and OB/GYN residents using means and standard deviations for normally distributed continuous variables, median and range for non-normally distributed continuous variables, and frequencies and percentages for categorical variables. To compare continuous variables across groups, parametric two-sample t-tests and non-parametric Mann–Whitney U tests were used for normally distributed and non-normally distributed variables, respectively. Shapiro–Wilk tests were used to assess the normality assumption in continuous variables. For group comparisons among categorical variables, chi-square or Fisher's exact tests were used, as appropriate.

To identify predictors of intentions to engage with CP, logistic regression models were estimated for intention to participate as a group facilitator in CP in their future practice and intention to talk with decision makers about establishing CP in future practice, respectively, stratified by different types of residents (Family Medicine and OB/GYN), adjusting for age, sex, race/ethnicity, residency year, and year of participation. Odds ratios, their 95% confidence intervals (CI), and corresponding *p*-values are presented. Statistical significance was concluded at the 0.05 level based on two-tailed tests. Stata/SE v15 was used for all data analyses [25].

## Results

In total, 186, 153, and 140 participants provided informed consent and completed the survey in 2017, 2018, and 2019, respectively. We did not collect data on residents who received the invitation but chose not to reply, thus we are unable to calculate the percentage of the total number of residents who responded to the survey. Participants whose professional status was reported as something other than an FM or OB/GYN resident (*n* = 2) and those who reported not intending to pursue obstetrics in future practice (*n* = 90) were removed from the analysis. Finally, a total of 388 participants (FM 54.64%; OB/GYN 46.36%) were included in analysis (150 in 2017, 127 in 2018, and 111 in 2019).

Demographic characteristics and residency year for responding residents are presented in Table 1. The majority of respondents in both OB/GYN (75.57%) and FM (70.14%) residencies were women and White. OB/GYN residents were younger than FM residents. Both FM and OB/GYN residents were, on average, in the third year of their residency training.

**Table 1:** Characteristics of respondents to a survey among U.S. Family Medicine and OB/GYN residents between 2017 to 2019

	**Type of residency**						
	**Overall**		**FM**		**OB/GYN**		***p*** ** value**
	***N*** ** = 388**		***N*** ** = 212**		***N*** ** = 176**		
Sex (n, %)							
Male	81	20.88	60	28.30	21	11.93	< 0.001^b^
Female	306	78.87	151	71.23	155	88.07	
Intersex	1	0.26	1	0.69	0	0	
Age (n, %)							
≤ 30	299	77.06	155	73.11	144	81.82	0.042^a^
> 30	89	22.94	57	26.89	32	18.18	
Race/Ethnicity (n, %)							
White	281	72.61	148	70.14	133	75.57	0.472^a^
Non-White	78	20.16	47	22.27	31	17.61	
Multiracial	28	7.24	16	7.58	12	6.82	
Residency year (mean, S.D)	2.19	0.99	2.12	0.88	2.27	1.11	0.063^c^

*SD* Standard Deviation

^a^Chi-square test

^b^Fisher's exact test

^c^Two-sample t-test

Descriptive statistics for predictor variables and outcomes are presented in Table 2. The level of engagement with CP reported by respondents was low (media*n* = 1; range:0–6). Respondents were most frequently exposed to CP through trainings held at the residency center (50.77%), followed by reading journal articles or other scholarly literature (21.65%), classroom study and formal coursework (20.62%), media attention (19.59%), continuing education through conferences or in-service training off-site (11.86%), and trainings held at the CenteringHealthcare Institute (8.25%). Only 31.70% of respondents received more than 15 h exposure to CP. Most of the respondents knew the accurate definition of CP as group-based, comprehensive prenatal care (87.63%). They reported high familiarity with CP (35.57% very familiar), and a high level of support for CP (media*n* = 23; range: 12–44). Meanwhile, they anticipated barriers to engaging with CP in their future practice (mea*n* = 24.96; SD: 4.87). Compared to OB/GYN residents, FM residents reported higher intention to participate as a group facilitator in CP in their future practice (59.66% and 73.11% respectively) and higher intention to talk with decision makers about establishing CP in their future practice (48.86% and 52.25%, respectively). Approximately 67.01% of the respondents intended to participate as a group facilitator in CP in their future practice and 51.80% of the respondents were willing to talk to decision makers about establishing CP in their future practice if the model is unavailable.

**Table 2:** CenteringPregnancy group prenatal care measures among U.S. Family Medicine (*N* = 212) and OB/GYN (*N* = 176) residents responding to a survey between 2017 to 2019

	**Type of residency**						
	**Overall**	**FM**		**OB/GYN**			***p*** ** value**
	***N*** ** = 388**	***N*** ** = 212**		***N*** ** = 176**			
***Predictors***							
Level of engagement (median, range)	1	0–6	1	0–6	1	0–6	0.293^d^
Types of engagement (n, %)							
Continuing education	46	11.86	28	13.21	18	10.23	0.366^a^
Trainings held at residency	197	50.77	119	56.13	78	44.32	0.020^a^
Trainings held at CHI	32	8.25	17	8.02	15	8.52	0.857^a^
Media attention	76	19.59	27	12.74	49	27.84	< 0.001^a^
Journal articles & scholarly literature	84	21.65	38	17.92	46	26.14	0.051^a^
Classroom and formal coursework	80	20.62	45	19.89	35	21.23	0.745^a^
Others	34	8.76	17	8.02	17	9.66	0.569^a^
Hours of exposure (n, %)							
< 5 h	147	37.89	81	38.21	66	37.50	0.115^a^
5–15 h	103	26.55	49	23.11	54	30.68	
> 15 h	123	31.70	76	35.85	47	26.70	
Knowledge (n, %)							
Haven't heard/ don’t know about it	19	4.90	12	5.66	7	3.98	0.60^b^
Support group/education class	28	7.22	16	7.55	12	6.82	
Group based comprehensive prenatal care*	340	87.63	184	86.79	156	88.64	
Familiarity (median, range)							
Not familiar	53	13.66	31	14.62	22	12.50	0.736^a^
Somewhat familiar	95	24.48	52	24.53	43	24.43	
Familiar	100	25.77	49	23.11	51	28.98	
Very familiar	138	35.57	79	37.26	59	33.52	
Level of support (median, range)	23	12–44	23	12–41	23	13–44	0.767^d^
Anticipated barriers (mean, S.D.)	24.96	4.87	25.07	4.86	24.83	4.90	0.316^c^
***Outcome***							
Intention to participate (n, %)							
Agree	260	67.01	155	73.11	105	59.66	< 0.001^a^
Intention to establish (n, %)							
Agree	201	51.80	115	52.25	86	48.86	0.025^a^

Intention to participate as a group facilitator in CenteringPregnancy (CP) in future practice and intention to talk to decision makers about establishing CP were scored 0 (did not agree) or 1 (agreed). Types of exposure was scored 0 – 6 with higher score corresponding to a higher exposure to continuing education through conferences or in-service training off-site, trainings held at the residency center, trainings held at the CenteringHealthcare Institute (CHI), classroom study and formal coursework, media attention (newspapers, radio, tv, social media, etc.), journal article or scholarly literature, or other source

*S.D* Standard Deviation

^*^The correct answer for the definition of CP

^a^Chi-square test

^b^Fisher's exact test

^c^Two-sample t-test

^d^nonparametric Mann–Whitney tests

Logistic regression model results for intention to participate as a group facilitator in CP if available in future practice, adjusting for covariates, are shown in Table 3. Among FM residents, those who received residency-based training on CP (adjusted Odds Ratio (aOR) 3.09, 95% CI 1.26, 7.54); spent more than 15 h engaged with CP (aOR 7.46, 95% CI 1.87, 29.79); had higher levels of support for CP (aOR 0.78, 95%CI 0.66, 0.92); and were more familiar with CP (aOR 5.74, 95%CI 1.08, 30.60) reported higher intention to participate as a group facilitator in CP in their future practice. Among OB/GYN residents, those who had higher levels of engagement with CP (aOR 1.40, 95%CI 1.02, 1.94); reported more than 15 h of exposure to CP (aOR 2.93, 95CI% 1.13, 7.63), and those who reported a higher level of support for CP (aOR 0.85, 95%CI 0.76, 0.96) were more likely to report an intention to participate as a group facilitator in CP in the future.

**Table 3:** The predictors of intention to participate as a group facilitator in CenteringPregnancy in their future practice, among U.S. Family Medicine (*N* = 212) and OB/GYN (*N* = 176) residents responding to a survey between 2017 to 2019, applying adjusted logistic regression models

	**FM**		**OB/GYN**	
	**Adjusted model**		**Adjusted model**	
	**O.R**	**95%CI**	**O.R**	**95%CI**
Level of engagement	1.43	0.92, 2.23	1.40*	1.02, 1.94
Type of engagement				
Continuing education off-site	7.18	0.89, 57.82	1.04	0.36, 3.05
Trainings held at residency	3.09*	1.26, 7.54	1.22	0.62, 2.42
Trainings held at CHI	3.03	0.37, 24.56	2.65	0.65, 10.83
Media attention	0.39	0.14, 1.07	1.45	0.68, 3.12
Journal articles & scholarly literature	0.95	0.35, 2.63	1.43	0.67, 3.07
Classroom study and formal coursework	1.25	0.47, 3.37	1.92	0.79, 4.67
Others	1.01	0.26, 3.97	1.69	0.44, 6.58
Hours of exposure				
< 5 h (ref)				
5–15 h	1.68	0.56, 4.98	1.10	0.49, 2.44
> 15 h	7.46**	1.87, 29.79	2.93*	1.13, 7.63
Familiarity				
Not familiar (ref)				
Somewhat familiar	0.97	0.24,3.84	1.12	0.33,3.83
Familiar	4.17	0.79,21.99	1.53	0.45,5.20
Very familiar	5.74*	1.08,30.60	2.63	0.74,9.31
Knowledge				
Haven't heard/ don’t know about it (ref)				
Support group/education class	1.80	0.18, 18.30	0.38	0.02, 6.52
Group based comprehensive prenatal care	5.73	0.68, 48.29	1.15	0.09, 14.95
Level of support	0.78**	0.66, 0.92	0.85**	0.76, 0.96
Anticipated barrier	1.03	0.95, 1.12	1.01	0.95, 1.08

Intention to participate as a group facilitator in CenteringPregnancy (CP) in their future practice was scored 0 (did not agree) or 1 (agreed). Types of exposure was scored 0 – 6 with higher score corresponding to a higher exposure to continuing education through conferences or in-service training off-site, trainings held at the residency center, trainings held at the CenteringHealthcare Institute (CHI), classroom study and formal coursework, media attention (newspapers, radio, tv, social media, etc.), journal article or scholarly literature, or other source. Covariates of the adjusted logistic regression models: year of participation, age, sex, year of residency training

*S.D* Standard Deviation, *O.R* Odds Ratio, *95%CI* 95% Confidence Interval

Significance: * *p* < 0.05, ** *p* < 0.01, *** *p* < 0.001

Logistic regression model results for intention to talk to decision makers about establishing CP if not available in future practice are presented in Table 4. Among FM residents, those who indicated a higher level of support for CP reported higher intention to talk to decision makers about establishing CP in their future practice (aOR 0.70, 95%CI 0.58, 0.85). Among OB/GYN residents, those who had higher levels of engagement with CP (aOR 1.56, 95CI% 1.15, 2.13); spent more than 15 h exposed to CP (aOR 2.72, 95%CI 1.11, 6.69), and who indicated more support for CP (aOR 0.81, 95% CI0.71, 0.93) reported higher intentions of talking with decision makers about establishing CP in their future practice.

**Table 4:** The predictors of intention to talk with decision makers about establishing CenteringPregnancy in future practice, among U.S. Family Medicine (*N* = 212) and OB/GYN(*N* = 176) residents responding to a survey between 2017 to 2019, applying adjusted logistic regression models

	**FM**		**OB/GYN**	
	**Adjusted model**		**Adjusted model**	
	**O.R**	**95%CI**	**O.R**	**95%CI**
Level of engagement	1.26	0.92, 1.75	1.56**	1.15, 2.13
Types of engagement				
Continuing education off-site	1.07	0.42,2.70	1.46	0.51,4.21
Trainings held at residency	1.44	0.71,2.93	1.63	0.84,3.18
Trainings held at CHI	1.26	0.40,4.02	1.05	0.34,3.24
Media attention	0.76	0.31,1.84	1.88	0.91,3.89
Journal articles & scholarly literature	1.7	0.72,4.03	1.96	0.94,4.08
Classroom and formal coursework	1.28	0.60,2.74	1.47	0.65,3.29
Others	2.19	0.64,7.50	1.8	0.57,5.67
Hours of exposure				
< 5 h (ref)				
5–15 h	1.06	0.44, 2.59	1.8	0.83, 3.92
> 15 h	2.15	0,78, 5.95	2.72*	1.11, 6.69
Familiarity				
Not familiar (ref)				
Somewhat familiar	0.9	0.28,2.89	0.6	0.18,2.00
Familiar	1.01	0.27,3.71	1.05	0.32,3.39
Very familiar	1.75	0.46,6.64	1.9	0.57,6.33
Knowledge				
Haven't heard/ don’t know about it (ref)				
Support group/education class	0.17	0.01, 2.31	0.31	0.02, 4.99
Group based comprehensive prenatal care	0.64	0.06, 6.81	0.73	0.06, 8.82
Level of support	0.70***	0.58, 0.85	0.81**	0.71, 0.93
Anticipated barrier	1.00	0.93, 1.07	1.04	0.97, 1.11

Intention to talk with decision makers about establishing CenteringPregnancy (CP) in their future practice was scored 0 (did not agree) or 1 (agreed). Types of exposure was scored 0 – 6 with higher score corresponding to a higher exposure to continuing education through conferences or in-service training off-site, trainings held at the residency center, trainings held at the CenteringHealthcare Institute (CHI), classroom study and formal coursework, media attention (newspapers, radio, tv, social media, etc.), journal article or scholarly literature, or other source

Covariates of the adjusted logistic regression models: year of participation, age, sex, year of residency training

*S.D* Standard Deviation, *O.R* Odds Ratio, *95%CI* 95% Confidence Interval

Significance: * *p* < 0.05, ** *p* < 0.01, *** *p* < 0.001

## Discussion

Experiences in residency with CP are predictors of residents’ intention and interest in offering CP in future practice. The majority of FM and OB/GYN residents who practice CP in residency intend to continue practicing this model of care if their future practice has an established CP program. Around half of respondents would plan to speak with decision makers about instituting it, which may attest to residents’ positive experiences and their belief that it is a good model of care. Engagement with CP, either in hours of exposure, level of engagement, or in the type of engagement, increased residents’ reported intention to practice the CP model in the future, with FM residents being particularly influenced by residency-based training on CP. Research indicates that providers appreciate what CP can offer in terms of high-quality care and enhanced role development [26]. It could be that training on CP in residency introduces FM residents to these concepts, motivating investment in the model. Obstetrics and gynecology residents reported less intention to facilitate CP in future practice than FM residents. This presents an important opportunity for OB/GYN residency programs to advocate for CP to their trainees. Future studies should examine the implementation process of incorporating CP into residency training and qualitatively explore why OB/GYN residents are less likely to advocate for GPNC than FM residents. There may be salient differences in implementation processes, as well as support among program administrators and among individual residents.

Level of support was more influential than anticipated barriers in predicting intention to engage with CP, suggesting that having higher levels of support toward the program may be key to developing the advocates necessary for the challenging realities of model implementation and enabling them to work toward long-term sustainability of the program. Novick et al. [24] identified attitudinal characteristics of staff who worked at facilities with thriving CP implementation. Providers’ motivation and buy-in were critical for addressing the barriers of implementation that can include structural problems, such as space, materials, or staffing, as well as problems with organizational culture, climate, and leadership. Positive opinions on change and innovation are also helpful in the successful adoption of novel programs [27].

Hours of exposure also had a positive impact on residents’ likelihood of practicing CP in the future. Physicians who offer CP have reported a rich, rewarding, and enjoyable professional experience where they perceived a higher quality of care and were able to get to know and support women [15]. In our study, the experience of engaging with CP in hours of exposure, type of engagement, or level of engagement appears to have a more lasting impact on intention than knowledge of the program on intention. Our findings indicate that while both FM and OB/GYN residents were not exposed to a wide variety of sources of information on CP (i.e. level of engagement), OB/GYN residents who had higher engagement with CP were more likely to report intention to talk with decision makers about establishing CP in the future. Diversifying ways for residents to engage with CP models may heighten their interest in and desire to be a champion for the program in the future.

The study has several limitations. First, while we reached out to residency programs across the United States, it is possible that respondents have differing views based on location, which should be explored in future studies. Second, our data does not reflect the overall resident population in the United States because we only collected data from residency programs that were operating a CP model of GPNC; residency programs that offer CP may differ from residency programs that do not offer CP in their willingness to try innovative programs. This could have led to self-selection bias. Third, because we did not collect data on how many residents were reached and who chose not to reply, we cannot calculate a response rate or conduct analyses examining for non-response bias. Fourth, an underlying assumption in all inferential analyses is that the responses are independent. However, it is possible that some residents may have responded to more than one survey over the data collection period. Because identifying information was not collected on the respondents, it is not possible to account for this dependence, or in other words, to control for within-subject effects. Finally, the residency program data was collected through an open-ended question, making it difficult to tally responses due to the variety of names different residents used when describing their residency. As a result, we were not able to control for the homogeneity effect within the same residency program during the analysis.

The strengths of this study include participation of residents across all years of study.

## Conclusions

Group prenatal care models such as CP can improve patient education, enhance social support, and produce positive obstetric outcomes [28]. Understanding resident physician perceptions of experiences with CP is an important step to learning how to effectively leverage residency to improve patient access to CP, especially in light of the challenges practitioners face implementing new models of care [29]. Our study provides evidence that engagement with and support towards CP during residency are key factors in FM and OB/GYN residents’ intention to practice CP in the future. In residency programs that provide CP for patients, program directors should consider increasing hours of exposure to CP and level of resident engagement with the model, particularly focusing on implementing residency-based training on CP among FM residents. Additionally, residents should be encouraged to participate in an open discussion of how to overcome potential barriers to implementing CP in their future practices.

### Supplementary Information

**Additional file 1.**

## Data Availability

The datasets used and/or analyzed during the current study available from the corresponding author on reasonable request.
