# Supplementary material for: National assessment of obstetrics and gynecology and family medicine residents’ experiences with CenteringPregnancy group prenatal care
Source: BMC Pregnancy Childbirth. 2023 Nov 21;23:805. doi: 10.1186/s12884-023-06124-0 (PMC10664296; doi:10.1186/s12884-023-06124-0)
Supplement: Supplementary file 1 — Additional file 1. [file 12884_2023_6124_MOESM1_ESM.docx]

**Appendix**

| **Social and Demographic Characteristics** | 1. What is your age?    1. Under 18    2. (options 19 through 65)    3. Over 65 2. What is your sex?    1. Male    2. Female    3. Intersex 3. What is your race/ethnicity (Mark as many as apply.)    1. White    2. Hispanic or Latino(a)    3. Black or African American    4. Native American or American Indian    5. Asian    6. Multiracial    7. Other 4. PG Year?    1. 1    2. 2    3. 3    4. 4    5. 5    6. 6 5. Name of Residency________________ 6. I am in a ______________ residency program.    1. Family medicine    2. OBGYN    3. Other (please specify) 7. What type of setting is your current residency practice?    1. Rural (less than 2,500 people)    2. Suburban (2,500 – 50,000 people)    3. Urban (more than 50,000 people)    4. Other (please specify) 8. I plan to do obstetrics in my future practice.    1. Yes    2. No    3. Unsure |
| --- | --- |
| **Intention to Engage with CenteringPregnancy in Future Practice** | 1. In relation to your future practice, please circle the response which best represents your opinions regarding the following statements. (strongly agree to strongly disagree)    1. I intend to participate as a group facilitator if CenteringPregnancy is available where I practice.    2. If CenteringPregnancy is not available where I practice, I intend to talk to decision makers about establishing it. |
| **Knowledge and Familiarity of CenteringPregnancy** | 1. CenteringPregnancy is __________________.    1. A support group    2. Group—based, comprehensive prenatal care    3. An educational class as adjunct to physician care    4. I’ve heard of CenteringPregnancy, but I’m unsure what it is    5. I’ve never heard of CenteringPregnancy 2. To what extent are you familiar with CenteringPregnancy? (1 not – 6 very) |
| **Experience with CenteringPregnancy** | 1. Have you completed training on CenteringPregnancy from the Centering Healthcare Institute?    1. Yes. If yes, what PG Year?    2. No 2. What type of exposure to CenteringPregnancy do you have? (mark as many as apply):    1. Attended CME (professional education, conference, or in-service off-site) where CenteringPregnancy was discussed    2. Attended informal training at my residency practice    3. Exposed to models of CenteringPregnancy through media attention (newspapers, radio, TV, social media, etc.)    4. Exposed through journal article or other scholarly literature    5. Exposed to CenteringPregnancy through classroom study and formal coursework    6. Exposed to CenteringPregnancy through other avenues (please describe) 3. Approximately how many hours do you think you have been exposed to CenteringPregnancy through any of the above experiences?    1. Less than 5 hours    2. 5-10 hours    3. 10-15 hours    4. More than 15 hours |
| **Level of Support for CenteringPregnancy** | 1. Please click the response which best represents your beliefs regarding the following statements. (strongly agree to strongly disagree)    1. CenteringPregnancy should be the standard of prenatal care (i.e. first option for prenatal care) in obstetric settings in the United States.    2. CenteringPregnancy should be an available option in all obstetric settings in the United States.    3. CenteringPregnancy provides more patient education than traditional one-on-one prenatal care.    4. CenteringPregnancy provides more patient-centered attention than traditional one-on-one prenatal care.    5. In CenteringPregnancy, providers have more time with their patients compared to traditional one-on-one prenatal care.    6. A benefit to CenteringPregnancy is the opportunity for women to provide social support to other women.    7. Women in CenteringPregnancy have more opportunities to involve their significant other, friends, or family members in their prenatal care compared to traditional one-on-one prenatal care.    8. The majority of women seeking prenatal care can receive high quality care appropriate to their individual needs within the CenteringPregnancy group model.    9. Nurse practitioners and certified nurse midwives are appropriate group facilitators for CenteringPregnancy. |
| **Anticipated Barriers** | 1. In relation to your future practice, please circle the response which best represents the challenges you might anticipate establishing CenteringPregnancy or being a physician at a practice that offers CenteringPregnancy.    1. I foresee productivity goals as a barrier to my engagement with CenteringPregnancy.    2. I foresee time management as a barrier to my engagement with CenteringPregnancy.    3. I foresee lack of institutional support as a barrier to establishing and maintaining CenteringPregnancy.    4. I foresee lack of appropriate training among office personnel as a barrier to establishing and maintaining CenteringPregnancy.    5. I foresee concerns about continuity of care for my patients as a barrier to my engagement with CenteringPregnancy.    6. I foresee concerns about quality of care as a barrier to my engagement with CenteringPregnancy.    7. I foresee concerns about costs to the practice as a barrier to my engagement with CenteringPregnancy. |
